# Memoranda on Difficult Subjects in Anatomy and Surgery; Being a Pocket Companion for Students Preparing for the College of Surgeons

**Published:** 1838-07

**Authors:** 


Art. XI.—1. *Memoranda on difficult subjects in Anatomy and Surgery, being a Pocket Companion for Students preparing for the College of Surgeons.* By Robert Druitt, m.r.c.s.l.—*London*, 1837. 48mo. pp. 184.

2. *The Student's Companion to Apothecaries' Hall; or the London Pharmacopœia of* 1836, *in Question and Answer.* By E. Oliver, m.r.c.s.—*London*, 1837. 48mo.

3. *Ophthalmic Memoranda, respecting those diseases of the Eye which are more frequently met with in Practice.* By John Foote, f.r.c.s.—*London*, 1838. 48mo. pp. 90.

4. *Libamenta Praxeos Medicæ, or a Manual of the Practice of Medicine, for the Use of Students and Junior Practitioners.* By D. Spillan, m.d.—*London*, 1838. 48mo. pp. 192.

5. *A Collection of Medical Formulæ, selected from the Writings of the most Eminent Physicians.* By D. Spillan, m.d.—*London*, 1838. 48mo. pp. 111.

We transcribe the preceding titles, not to attract attention to the literary abortions to which they are prefixed, but to warn all and sundry our younger readers who might be seduced by their exterior, to eschew them, as beneath the notice of every honorable student, who is conscious of a desire to study medicine in the spirit which so noble a profession should call forth. Notwithstanding the suspicious circumstance of the almost simultaneous appearance of these Lilliputian manuals for Lilliputian minds, we cannot bring ourselves for a moment to swerve from the opinion which we have gladly cherished, that our London students are progressively improving in their general character of attention to their studies, and in their disposition to learn medicine and surgery thoroughly and soundly. We are inclined to attribute the sudden outbreak of this epidemic of infinitesimal manuals and guides, rather to the teeming fancy of some hopeful publisher, than to the actual demand for such ware in the present state of the medical market. We strongly deprecate all such publications: they pander to the depraved taste of the ignorant and the idle, while their very existence is a sort of slander on the industrious and well-informed. The sole good we anticipate from them is that, by attracting the notice of the examiners at the different colleges, halls, and boards, they may induce these gentlemen to make their examinations still more searching and complete, and thus sift the honest students, the men of real knowledge, from the disgraceful fry—if there are indeed such—who have been stuffed for the nonce by Grinders and Guide-books.

